# Age-Stratified Association Between CPI Screening Categories and Caries Experience in the Permanent Dentition of Children and Adolescents from Southwest Romania: A Multicenter Cross-Sectional Clinical Study

**DOI:** 10.3390/children13081060

**Published:** 2026-08-10

**Authors:** Narcis Mihaiță Bugălă, Adelina Smaranda Bugălă, Mihaela Jana Țuculină, Dana Maria Albulescu, Alina Nicoleta Căpitănescu, Dragoș Cadea, Adrian Macovei, Dragoș Alexandru, Ana Maria Rîcă, Ancuța Ramona Camen

**Affiliations:** 1 Doctoral School, University of Medicine and Pharmacy of Craiova, 200349 Craiova, Romania; bugala.mihai@yahoo.com; 2 Department of Endodontics, Faculty of Dental Medicine, University of Medicine and Pharmacy of Craiova, 200349 Craiova, Romania; r_ana_maria22@yahoo.com; 3 Department of Anatomy, Faculty of Medicine, University of Medicine and Pharmacy of Craiova, 200349 Craiova, Romania; med73danam@yahoo.com (D.M.A.); dragos.cadea@umfcv.ro (D.C.); adrian.macovei@umfcv.ro (A.M.); 4 Department of Otorhinolaryngology, Faculty of Medicine, University of Medicine and Pharmacy of Craiova, 200349 Craiova, Romania; alinacapitanescu@yahoo.com; 5 Department of Biostatistics, Faculty of Medicine, University of Medicine and Pharmacy of Craiova, 200349 Craiova, Romania; dragos.alexandru@umfcv.ro; 6 Department of Occupational Medicine, Faculty of Medicine, University of Medicine and Pharmacy of Craiova, 200349 Craiova, Romania; ancutzaboicea@gmail.com

**Keywords:** dental caries, DMF-T, Community Periodontal Index, Gingival Index, Plaque Index, children, adolescents, cross-sectional study

## Abstract

**Highlights:**

**What are the main findings?**

- Higher Community Periodontal Index (CPI) screening categories were consistently associated with greater permanent-dentition caries experience (DMF-T) in both children (6–12 years) and adolescents (13–19 years).
- The CPI–DMF-T association remained consistent across age groups, although adjustment for Plaque Index substantially attenuated the observed associations, indicating considerable overlap between plaque accumulation and CPI findings.

**What is the implication of the main finding?**

- Unfavorable Community Periodontal Index (CPI) findings may prompt a broader oral-health assessment in clinical practice and school-based prevention programs.
- Prospective longitudinal studies are required before CPI can be considered an independent predictor of future caries development.

**Abstract:**

Background/Objectives: Dental caries and gingival inflammation share several biofilm-related determinants. This study evaluated the association between Community Periodontal Index (CPI) screening categories and permanent-dentition caries experience in a consecutively recruited clinical sample of children and adolescents from Southwest Romania. Methods: The multicenter cross-sectional study included 638 participants aged 6–19 years, analyzed as 6–12 years (n = 407) and 13–19 years (n = 231). Analyses included nonparametric comparisons, adjusted Poisson models, a formal CPI-by-age-stratum interaction, continuous-age sensitivity analyses, and generalized Poisson models allowing underdispersion. Results: Mean DMF-T was 4.71 ± 2.55 and 4.93 ± 2.58 in the younger and older strata, respectively. CPI correlated strongly with DMF-T (rho = 0.743 and 0.749; both *p* < 0.001). Adjusted expected DMF-T counts were higher for CPI 1 and CPI 2 than for CPI 0 in both strata. There was no statistical evidence that the CPI–DMF-T association differed by age stratum (global interaction *p* = 0.749) or continuous age (*p* = 0.552). Plaque Index adjustment markedly attenuated CPI estimates; the extent of residual CPI association was sensitive to count-model specification. Conclusions: Higher CPI categories were associated with greater cumulative caries experience in this clinical sample, but the findings do not establish temporality, causality, or predictive validity. Unequal numbers of erupted permanent teeth and CPI-eligible sextants may have contributed to the observed gradient because these quantities were not retained analytically.

## 1. Introduction

Dental caries and plaque-induced gingival inflammation are common oral conditions during childhood and adolescence. Although they have distinct clinical outcomes, both are strongly influenced by the persistence of dental biofilm and by related behavioral and social determinants, including frequent free-sugar intake, inadequate plaque control, insufficient fluoride exposure, irregular dental attendance, and socioeconomic disadvantage [1,2,3,4,5,6,7,8,9,10,11,12,13,14,15]. Their coexistence is therefore biologically plausible, but the relationship should not be interpreted as a direct causal effect of gingival inflammation on caries development. Rather, caries experience and gingival inflammatory findings may represent different clinical manifestations of a shared unfavorable oral health environment [13,14,15].

The Community Periodontal Index (CPI) provides a standardized method for recording selected periodontal findings, particularly bleeding on probing and calculus. In children and adolescents, CPI should be regarded as a screening indicator rather than as a comprehensive periodontal diagnostic instrument, because it does not fully characterize gingival inflammation, attachment loss, disease extent, or periodontal stability [14,15,16]. Nevertheless, higher CPI screening categories may identify individuals with persistent plaque accumulation and inadequate oral hygiene. These conditions may also favor caries development through prolonged biofilm retention and repeated exposure of susceptible tooth surfaces to a cariogenic environment. An association between CPI and the Decayed, Missing and Filled Teeth index (DMF-T) would therefore be expected to reflect shared biofilm-related and behavioral determinants rather than an independent pathogenic effect of periodontal status on caries.

Available Romanian studies indicate a substantial but heterogeneous burden of dental caries among children. A multicenter study of Romanian schoolchildren reported caries experience in 84.3% of children aged 6–8 years and 83.1% of those aged 11–13 years, although estimates varied according to the diagnostic threshold applied [17].

In a survey of 12-year-old children from southern and central Romania, 63.3% had caries experience and the mean DMF-T score was 2.89 [18]. A separate study of Romanian children aged 6–8 years described dental caries in relation to oral-health behaviors and living conditions [19].

Other regional investigations reported substantially higher values, including a caries prevalence of 95.5% and a mean DMF-T score of 3.13 among Romanian adolescents [20].

These differences probably reflect variation in participants’ ages, diagnostic criteria, geographic setting, and sampling procedures, and they limit direct comparison between studies.

Regional and residential inequalities have also been identified. In a national school-based investigation, the proportion of caries-free children in Oltenia was 17.81% in rural areas and 26.32% in urban areas, while the number of restorations remained low in this region [21]. Broader European evidence also indicates that area-level factors contribute to inequalities in childhood and adolescent caries [22].

A separate Romanian survey found differences in lesion patterns and treatment experience between urban and rural children, including a greater frequency of restorations in urban participants and more advanced untreated lesions in some rural groups [23].

These findings suggest that place of residence may reflect differences in access to preventive and restorative care, although the direction and magnitude of urban–rural disparities have not been consistent across all Romanian samples [20].

Data concerning gingival health are more limited and are concentrated in a small number of regional studies. A study of schoolchildren in Bucharest reported gingivitis in 91% of participants, predominantly as mild inflammation, and found poorer gingival conditions among children exposed to less favorable household and educational circumstances [24].

More recent Romanian data similarly indicate that visible plaque and gingival inflammation remain frequent among schoolchildren, despite commonly reported toothbrushing [25].

However, Romanian studies have generally evaluated caries experience, oral hygiene, or gingival status separately, in restricted age groups or specific geographic settings. Evidence directly examining the graded relationship between CPI screening categories and caries experience across childhood and adolescence remains limited, particularly in Southwest Romania.

Accordingly, the primary objective of this cross-sectional study was to evaluate the age-stratified association between participant-level CPI screening category and permanent-dentition caries experience, expressed by the DMF-T score, in children aged 6–12 years and adolescents aged 13–19 years from Southwest Romania. Secondary objectives were to compare the prevalence of caries experience and DMF-T distributions across CPI categories within each age stratum, to estimate adjusted categorical and ordinal CPI associations after accounting for age, sex, area of residence, and county, and to examine how the CPI estimates changed after separate adjustment for Plaque Index and Gingival Index.

## 2. Materials and Methods

### 2.1. Study Design, Setting, and Recruitment

This multicenter cross-sectional clinical study was conducted between April 2025 and April 2026 in Southwest Romania. Recruitment was performed in four dental care settings: one school dental office in Gorj County, one school dental office in Olt County, and two centers in Dolj County, comprising one school dental office and the Endodontics Clinic of the Faculty of Dentistry, University of Medicine and Pharmacy of Craiova. All participating centers provided dental examination or treatment services to children and adolescents.

Participants were patients who attended the participating dental facilities for clinical examination or treatment during the study period. A consecutive, non-probability sampling method was used. All patients presenting during the recruitment period were screened for eligibility and were invited to participate when the predefined inclusion criteria were met. No recruitment quotas or predefined numerical targets were established for individual counties or centers. Recruitment proceeded consecutively during the study period, and the near-equal final numbers observed across the three counties occurred incidentally. Center identifiers and center-specific screening logs were not retained in the final analytical database; consequently, center-specific participant-flow counts and fixed-effect adjustment for recruitment center could not be reconstructed.

A total of 674 individuals were assessed for eligibility. Thirty-six were excluded because they did not meet one or more of the predefined eligibility criteria, and 638 participants were included in the final analysis.

Because participants were recruited among patients attending dental services rather than through a population-based sampling frame, the analytical sample was considered a clinical sample and was not assumed to be representative of all children and adolescents living in Southwest Romania.

The study was reported in accordance with the STROBE recommendations for observational cross-sectional studies [26].

### 2.2. Pragmatic Sample Size Estimation

The study used a pragmatic sample size approach. The initial calculation was intended to ensure reasonable precision for the estimation of oral-health prevalence indicators within the clinical sample rather than to provide formal statistical power for the primary association model.

Using a 95% confidence level, an expected proportion of 50%, selected as the most conservative assumption, and an absolute precision of 4%, the estimated minimum sample size was 600 participants. After allowing approximately 5% for incomplete or unusable clinical records, the recruitment target was set at no fewer than 630 participants. The final analytical sample comprised 638 participants.

### 2.3. Eligibility Criteria

Participants were eligible if they were aged 6–19 years, attended one of the participating dental facilities during the recruitment period, had at least one CPI-eligible sextant containing a minimum of two fully erupted and clinically assessable permanent teeth, and were able to undergo the standardized oral examination. Only permanent teeth were included in all dental and periodontal study assessments. Written informed consent was provided by a parent or legal guardian for participants younger than 18 years, together with age-appropriate assent where applicable. Participants aged 18 years or older provided their own written informed consent.

Exclusion criteria were fixed orthodontic treatment; severe dento-maxillary anomalies preventing standardized examination; acute oral conditions requiring immediate treatment before completion of the study examination; systemic diseases or medication use known to substantially alter gingival or periodontal findings, including disorders associated with abnormal bleeding, marked immune dysfunction, or gingival enlargement; professional periodontal treatment or systemic antibiotic use within the previous three months; inability to cooperate with the clinical examination; and incomplete clinical records or missing data for the primary study variables.

### 2.4. Dental Caries Assessment

Dental caries experience was assessed exclusively in the permanent dentition using the Decayed, Missing, and Filled Teeth index for permanent teeth, reported as DMF-T, according to World Health Organization criteria [16]. Primary teeth were not included in the study indices, and no dmft score was calculated.

Only fully erupted permanent teeth that could be examined reliably were included. Partially erupted permanent teeth were excluded from DMF-T assessment. Teeth that could not be examined adequately because of limited clinical access, extensive soft-tissue coverage, or other local conditions were recorded as not assessable.

Caries was diagnosed clinically by visual-tactile examination under artificial light, using a plane dental mirror and a blunt-ended probe. Radiographs were not used for study classification.

The DMF-T score constituted the primary outcome. The presence of caries experience, defined as DMF-T > 0, was treated as a secondary outcome for prevalence comparisons across CPI categories. Teeth missing for reasons unrelated to caries, including orthodontic extraction, trauma, or congenital absence, were not included in the M component. No analytic upper limit was imposed on DMF-T; the observed maximum value of 10 arose naturally from the recorded data. Third molars were excluded from CPI assessment; however, their contribution to the final participant-level DMF-T variable was not documented separately in the analytical database.

### 2.5. Community Periodontal Index Assessment

Gingival and periodontal screening findings were recorded using a full-mouth CPI-based protocol adapted from World Health Organization principles [16]. The permanent dentition was divided into six sextants: maxillary right posterior, maxillary anterior, maxillary left posterior, mandibular left posterior, mandibular anterior, and mandibular right posterior. Third molars were excluded.

All fully erupted and clinically assessable permanent teeth were examined. Partially erupted permanent teeth were excluded. Six sites were assessed for each eligible tooth: mesiobuccal, mid-buccal, distobuccal, mesiolingual or mesiopalatal, mid-lingual or mid-palatal, and distolingual or distopalatal. A WHO periodontal probe was used with light probing pressure.

A sextant was considered eligible when it contained at least two fully erupted and clinically assessable permanent teeth. Sextants containing fewer than two eligible permanent teeth were recorded as ineligible and were excluded from sextant-level scoring. Primary teeth were not used as substitutes for permanent teeth and did not contribute to CPI assessment.

For each eligible sextant, the highest score identified at any examined site was recorded: CPI 0, no bleeding on probing and no detectable calculus; CPI 1, bleeding on probing without detectable calculus; and CPI 2, supra- or subgingival calculus detected during probing. The participant-level CPI category was defined as the highest CPI score recorded across all eligible sextants.

The complete CPI coding framework was considered during the clinical examination. However, no findings corresponding to CPI codes 3 or 4 were recorded in this pediatric and adolescent sample. Consequently, the participant-level analytical variable comprised the three observed categories: CPI 0, CPI 1, and CPI 2. Participants without at least one eligible sextant were considered ineligible for the study rather than excluded only from the CPI analysis.

In participants with mixed dentition, the CPI protocol was applied exclusively to fully erupted permanent teeth. Primary teeth, exfoliating teeth, and partially erupted permanent teeth were excluded from scoring.

### 2.6. Gingival Index and Plaque Index Assessment

Gingival inflammation and plaque accumulation were assessed according to the Gingival Index described by Löe and Silness and the Plaque Index described by Silness and Löe [27,28]. For each fully erupted and clinically assessable permanent tooth, four surfaces were evaluated: distal, facial or buccal, mesial, and lingual or palatal. Each surface was assigned a score ranging from 0 to 3 according to the original criteria of the respective index.

For each tooth, the Gingival Index and Plaque Index scores were calculated as the arithmetic mean of the corresponding surface scores. Participant-level Gingival Index and Plaque Index values were subsequently calculated as the arithmetic mean of the tooth-level scores across all fully erupted and clinically assessable permanent teeth. Thus, the values used in the statistical analyses represented continuous participant-level mean scores rather than directly assigned global ordinal categories.

In participants with mixed dentition, the calculation was restricted to fully erupted and clinically assessable permanent teeth. Primary teeth, exfoliating teeth, and partially erupted permanent teeth were excluded.

The surface-level scores were used to calculate the tooth-level and participant-level indices but were not retained as separate variables in the final analytical database.

### 2.7. Examiner Calibration and Data Quality

All clinical examinations were performed by a single examiner. Before participant recruitment, the examiner underwent theoretical and clinical training in the application of the DMF-T, CPI, Gingival Index, and Plaque Index criteria.

Intra-examiner quality control involved repetition of the complete oral examination in a randomly selected subsample of 62 participants after 7–10 days, with the examiner blinded to the initial findings. A historical pooled Cohen’s kappa coefficient of 0.86 was calculated during data collection. However, the paired index-specific reassessment dataset and the unit-level structure used for that pooled calculation were not retained. The pooled coefficient is therefore not interpreted as evidence of reproducibility for DMF-T, CPI, Gingival Index, or Plaque Index individually, and index-specific weighted kappa or intraclass correlation coefficients could not be reconstructed.

Clinical forms were reviewed for completeness before database entry. The database was subsequently checked for duplicate records, values outside the permitted ranges, internal inconsistencies, and coding errors. Participants were identified in the analytical database using unique numerical study codes, and directly identifying information was not included in the statistical dataset.

### 2.8. Statistical Analysis

Statistical analyses were performed using JASP version 0.96.0 (JASP Team, University of Amsterdam, Amsterdam, The Netherlands). The Descriptives, Frequencies, ANOVA/nonparametric, Regression, and Generalized Linear Model procedures were used. All tests were two-sided, and *p* < 0.05 was considered statistically significant. The 6–12-year and 13–19-year strata were defined a priori for the present analysis to distinguish mixed-dentition childhood from predominantly permanent-dentition adolescence and to reduce heterogeneity related to dental development. Because the clinical protocol assessed only fully erupted permanent teeth, DMF-T was retained as the caries outcome in both strata and was not pooled with a primary-dentition dmft index.

Continuous variables were summarized using means and standard deviations, medians and interquartile ranges, and minimum-maximum values. Categorical variables were summarized using frequencies and percentages. There were no missing values for the principal analytical variables.

Associations between DMF-T and CPI, Plaque Index, Gingival Index, and age were evaluated using Spearman rank correlation. Nonparametric bootstrap 95% confidence intervals based on 3000 participant-level resamples were calculated for Spearman coefficients. DMF-T distributions across CPI categories were compared using the Kruskal–Wallis test. Rank epsilon squared with 95% confidence intervals was reported as the effect-size measure, and Dunn pairwise comparisons were adjusted using the Holm procedure.

The prevalence of caries experience, defined as DMF-T > 0, was compared across CPI categories using Pearson chi-square testing and Cramer’s V in the 6–12-year stratum. In the 13–19-year stratum, sparse expected counts required an exact comparison of CPI 0 versus CPI ≥ 1 using Fisher’s exact test and the phi coefficient. Bootstrap 95% confidence intervals were calculated for Cramer’s V and phi.

Primary adjusted analyses used Poisson generalized linear models with a logarithmic link. CPI was first modeled as a categorical factor, with CPI 0 as the reference category, and the models were adjusted for age, sex, area of residence, and county. Exponentiated coefficients are reported as expected DMF-T count ratios, avoiding incidence terminology in this cross-sectional analysis. A secondary trend model coded CPI ordinally as 0, 1, and 2. Relative fit of categorical and ordinal specifications was compared using the change in deviance.

Separate contextual Poisson models added either Plaque Index or Gingival Index to the categorical CPI model. The two indices were not entered simultaneously because of their high correlation. PI and GI coefficients were scaled to represent a clinically interpretable 0.5-unit increase. Model fit and diagnostics included likelihood-ratio tests, Akaike and Bayesian information criteria, deviance, Pearson chi-square divided by residual degrees of freedom, variance-inflation factors, standardized residuals, and Cook’s distance. A combined model containing CPI, age stratum, and their interaction formally tested effect modification. Sensitivity analyses treated age continuously, included a quadratic age term, and tested CPI-by-continuous-age interactions. Histograms, violin/boxplots with individual observations, and jittered scatterplots were used to examine outcome distributions and the relationships of DMF-T with PI and GI.

## 3. Results

### 3.1. Participant Flow

Between April 2025 and April 2026, 674 children and adolescents were assessed for eligibility across the four participating dental care settings. Thirty-six individuals (5.3%) were excluded because they did not meet one or more predefined eligibility criteria, and 638 participants (94.7%) entered the analysis. Center-specific screening, exclusion, and inclusion counts and the individual reasons for the 36 exclusions were not retained in the analytical database and could not be reconstructed. Overall participant flow is presented in Supplementary Figure S4.

All included participants had at least one CPI-eligible sextant containing a minimum of two fully erupted and clinically assessable permanent teeth. Only permanent teeth were included in the DMF-T, CPI, Gingival Index, and Plaque Index assessments.

### 3.2. Participant Characteristics

The sample included 407 children aged 6–12 years and 231 adolescents aged 13–19 years. There were no missing values for the principal variables. Demographic and clinical characteristics are summarized in Table 1.

### 3.3. Caries Experience Across CPI Categories

**Figure 1:**
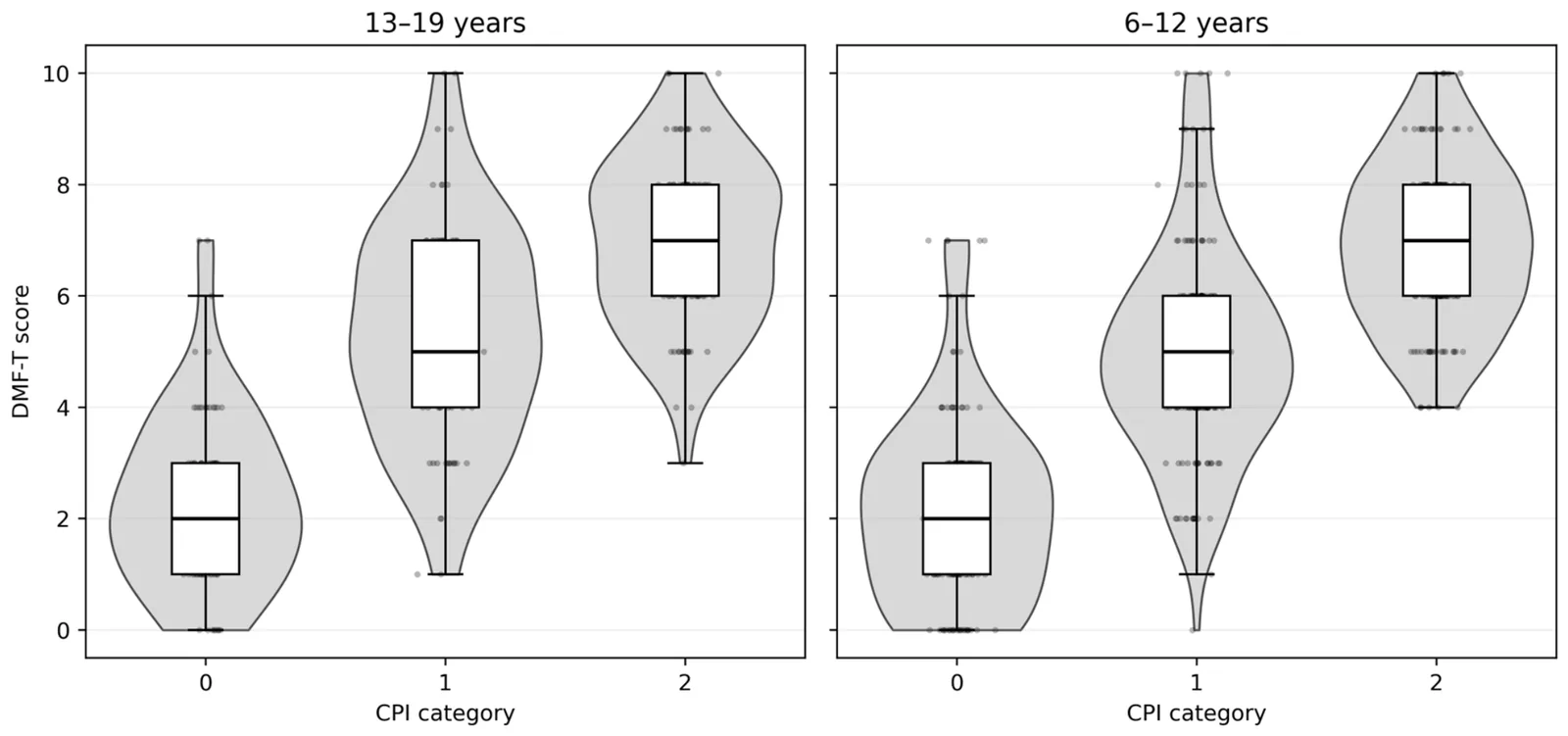
Permanent-dentition DMF-T distributions across CPI categories by age stratum. Violin shapes show the observed distributions, internal boxplots show medians and interquartile ranges, and points represent individual participants.

The prevalence of caries experience and DMF-T distributions showed clear gradients across CPI categories in both age strata. Among children aged 6–12 years, prevalence increased from 82.7% in CPI 0 to 99.4% in CPI 1 and 100.0% in CPI 2 (chi-square(2) = 47.22, *p* < 0.001; Cramer’s V = 0.341, 95% CI: 0.254–0.417). Among adolescents aged 13–19 years, prevalence was 89.5% in CPI 0 and 100.0% in CPI 1 and CPI 2. Because expected counts were sparse, CPI 1 and CPI 2 were combined for exact testing (Fisher’s exact *p* < 0.001; phi = 0.270, 95% CI: 0.167–0.359). The distributional gradients are displayed using violin/boxplots with individual observations in Figure 1; age-stratified DMF-T histograms are provided in Supplementary Figure S1. Detailed age-stratified prevalence and DMF-T estimates are presented in Table 2.

Spearman correlations confirmed strong associations between CPI and DMF-T in both strata (rho = 0.743, 95% CI: 0.693–0.787, and rho = 0.749, 95% CI: 0.686–0.801; both *p* < 0.001). DMF-T was also strongly associated with Plaque Index (rho = 0.880, 95% CI: 0.856–0.898, and rho = 0.876, 95% CI: 0.843–0.902) and Gingival Index (rho = 0.847, 95% CI: 0.820–0.869, and rho = 0.837, 95% CI: 0.794–0.866; all *p* < 0.001). Age was not significantly correlated with DMF-T. Kruskal–Wallis testing demonstrated large between-category differences in both strata, and all Dunn–Holm pairwise comparisons were significant (all adjusted *p* < 0.001). Jittered scatterplots are presented in Supplementary Figures S2 and S3. Correlation and Kruskal–Wallis results are summarized in Table 3.

### 3.4. Adjusted Poisson Models

The primary categorical CPI models were statistically significant in both age strata. Compared with CPI 0, the adjusted expected DMF-T count was 2.27 times as high for CPI 1 and 3.16 times as high for CPI 2 among children aged 6–12 years. Among adolescents aged 13–19 years, the corresponding expected count ratios were 2.28 and 2.99. Age, sex, area of residence, and county were not statistically associated with DMF-T after mutual adjustment. The complete adjusted estimates are presented in Table 4.

The Poisson models were underdispersed. For ages 6–12 years, likelihood-ratio chi-square(7) = 331.9, AIC = 1647, BIC = 1679, deviance/df = 0.87, and Pearson chi-square/df = 0.80. For ages 13–19 years, likelihood-ratio chi-square(7) = 198.2, AIC = 935.2, BIC = 962.7, deviance/df = 0.77, and Pearson chi-square/df = 0.70. Diagnostic review identified no influential observations with material Cook’s distances.

**Figure 2:**
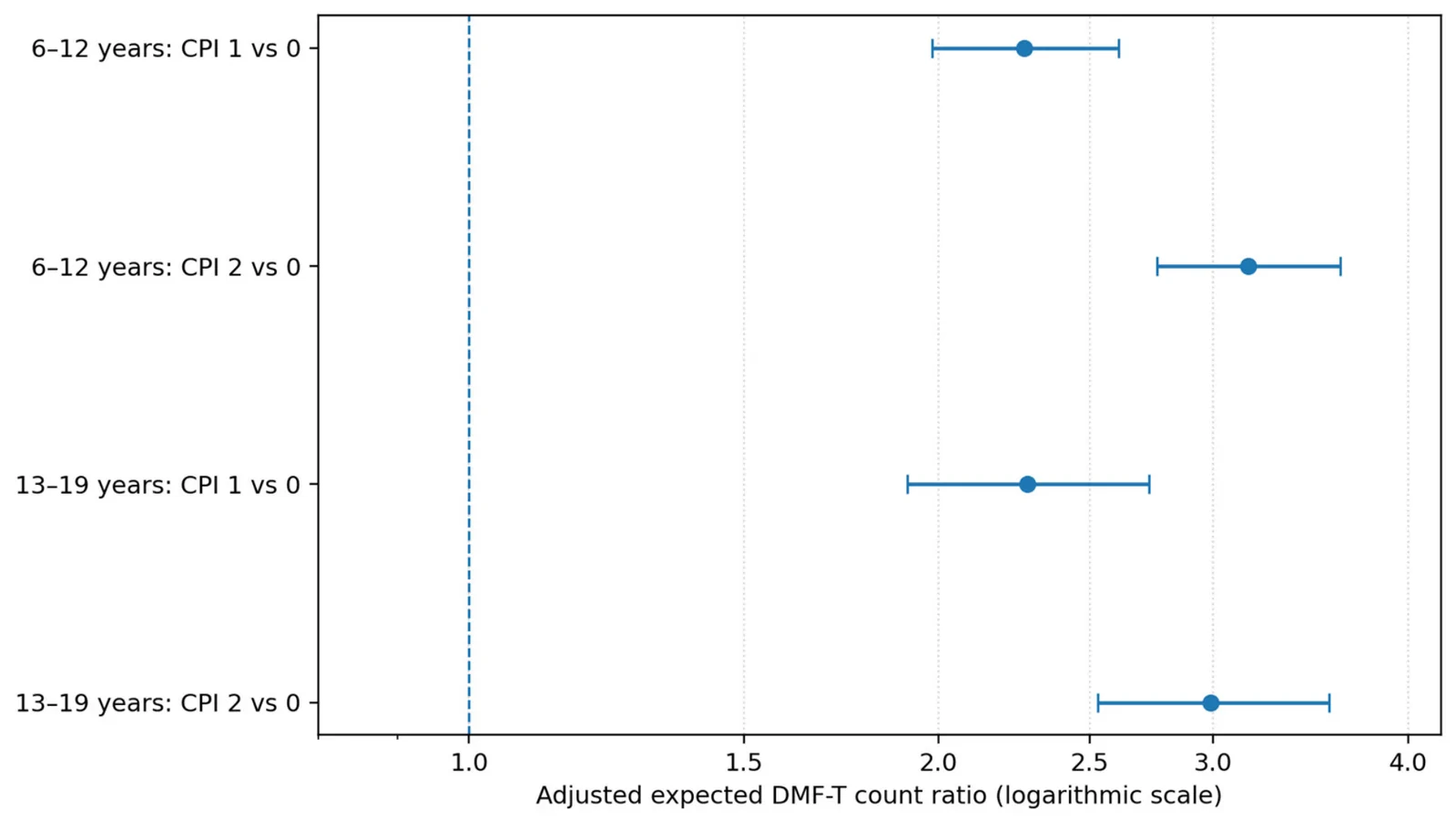
Adjusted expected DMF-T count ratios for CPI categories from the primary Poisson models. Models were adjusted for age, sex, area of residence, and county.

The ordinal trend models were also significant. Each one-category CPI increase was associated with a 68.5% higher expected DMF-T count in the younger stratum (expected count ratio = 1.69, 95% CI: 1.59–1.79) and a 63.9% higher expected count in the older stratum (1.64, 95% CI: 1.52–1.77; both *p* < 0.001). However, the categorical models fitted better than the trend models (younger: delta deviance = 24.2, df = 1; older: delta deviance = 16.5, df = 1; both *p* < 0.001), indicating that the increase was not strictly linear across CPI categories. In the combined adjusted model, neither CPI-by-age-stratum interaction was significant (CPI 1 interaction ratio = 0.99, 95% CI: 0.79–1.24, *p* = 0.951; CPI 2 interaction ratio = 1.05, 95% CI: 0.85–1.31, *p* = 0.637), and the global interaction test was not significant (likelihood-ratio chi-square(2) = 0.58, *p* = 0.749). This indicates no statistical evidence of effect modification by age stratum, not proof of equivalence. Adjusted CPI count ratios from these models are shown in Figure 2.

### 3.5. Contextual Models with Plaque Index and Gingival Index

Plaque Index and Gingival Index were highly correlated in both age strata (Spearman’s rho = 0.894 in participants aged 6–12 years and 0.906 in those aged 13–19 years; both *p* < 0.001) and were therefore included in separate contextual models. In children aged 6–12 years, each 0.5-unit PI increase was associated with a 35% higher expected DMF-T count; CPI 1 remained significant, whereas CPI 2 did not. Among adolescents, each 0.5-unit PI increase was associated with a 29% higher expected count, and neither CPI category was significant in the conventional Poisson model after PI adjustment. In the GI-adjusted models, each 0.5-unit GI increase was associated with 30% and 27% higher expected counts in the younger and older strata, respectively, and both CPI categories remained significant. Complete coefficients for all demographic and geographic covariates are provided in Supplementary Table S1. The PI models had lower AIC values than the corresponding GI models. The contextual model estimates are summarized in Table 5.

### 3.6. Sensitivity Analyses and Diagnostics

When age was modeled continuously with a quadratic term, neither the linear nor quadratic age term was statistically significant. CPI-by-continuous-age interactions were also non-significant (CPI 1 interaction ratio = 0.99, 95% CI: 0.95–1.02, *p* = 0.397; CPI 2 interaction ratio = 0.98, 95% CI: 0.95–1.02, *p* = 0.277), and the global interaction did not improve fit (likelihood-ratio chi-square(2) = 1.19, *p* = 0.552). The results were therefore not materially dependent on the boundary between 12 and 13 years.

Generalized Poisson sensitivity analyses estimated negative dispersion parameters in every model, confirming underdispersion. The primary CPI associations remained strong and directionally similar: expected count ratios were 2.20 and 3.04 in the 6–12-year stratum and 2.19 and 2.85 in the 13–19-year stratum for CPI 1 and CPI 2, respectively (all *p* < 0.001). In PI-adjusted sensitivity models, CPI 2 remained non-significant among children, whereas both adolescent CPI contrasts had narrower confidence intervals that excluded the null. Thus, the primary CPI association was robust, but the evidence for an additional CPI association after PI adjustment was sensitive to the count-distribution specification. Full sensitivity results are provided in Supplementary Table S2.

## 4. Discussion

The present analysis identified a strong association between CPI screening categories and permanent-dentition caries experience in both age strata. The descriptive gradients, strong rank correlations, large Kruskal–Wallis effect sizes, and adjusted Poisson models all supported this relationship. Formal interaction testing found no statistical evidence that the CPI–DMF-T association differed between the 6–12-year and 13–19-year strata, and the continuous-age sensitivity analysis reached the same conclusion. These findings demonstrate consistency within this clinical sample but should not be interpreted as proof that the age-specific effects are equivalent. Previous pediatric and adolescent studies have also reported relationships among plaque accumulation, gingival or periodontal findings, caries experience, and related behavioral or sociodemographic factors, although findings have varied across populations [29,30,31,32,33,34,35,36,37,38,39,40].

Mean DMF-T increased from 2.20 in CPI 0 to 4.99 in CPI 1 and 6.93 in CPI 2 among participants aged 6–12 years; corresponding adolescent values were 2.32, 5.33, and 6.98. Recent European school-based studies show substantially more heterogeneous caries burdens than the present clinical sample. Randomly sampled Swiss schoolchildren showed age- and sociodemographic variation in caries experience [6], while Polish adolescents continued to exhibit substantial DMF-T associated with oral-health behaviors [39]. A recent European neighborhood review similarly documented marked geographic and socioeconomic heterogeneity [22]. These contrasts reinforce that the prevalence exceeding 94% in our study reflects a care-seeking clinical sample and should not be interpreted as a population estimate for Southwest Romania.

The observed association should not be interpreted as evidence that gingival inflammation, calculus, or plaque accumulation caused the cumulative DMF-T experience. DMF-T combines untreated decay, teeth missing because of caries, and previous restorative treatment, whereas CPI, PI, and GI were recorded contemporaneously. The measures therefore represent partly different time periods. They share plausible determinants—including persistent biofilm, free-sugar exposure, fluoride exposure, oral-hygiene practices, dental attendance, and socioeconomic circumstances—but temporal ordering cannot be established in this cross-sectional design [8,9,10,11,12,13,14,15].

The contextual models qualify the primary association. PI adjustment substantially attenuated the CPI coefficients. In the conventional Poisson models, CPI 2 was not significant in the younger stratum and neither adolescent CPI category remained significant after PI adjustment. This represents an absence of evidence for an additional CPI association beyond PI within those models. Generalized Poisson sensitivity analyses produced similar point estimates but narrower intervals; both adolescent CPI contrasts then excluded the null. Accordingly, the residual CPI association after PI adjustment is model-sensitive. The most defensible interpretation is that CPI and PI contain substantial overlapping information about the contemporaneous oral environment, not that PI mediates the association or that CPI predicts future caries.

When Gingival Index was introduced instead of Plaque Index, GI and both CPI categories remained statistically significant in both age strata. The PI models nevertheless had lower AIC values than the corresponding GI models. This difference may reflect the more direct measurement of plaque accumulation by PI, whereas GI summarizes the host inflammatory response. Because all variables were measured at the same examination, neither attenuation nor persistence of coefficients establishes mediation, causality, or predictive validity.

Plaque Index and Gingival Index were analyzed in separate models because they were highly correlated in both age strata. This modeling strategy was chosen to avoid potentially unstable estimates resulting from the simultaneous inclusion of two highly correlated clinical indicators. The different behavior of CPI in the PI-adjusted and GI-adjusted models also shows that these measures are related but not interchangeable. CPI records the most unfavorable bleeding- or calculus-related finding across eligible sextants, whereas participant-level PI and GI summarize mean plaque accumulation and gingival inflammation across the examined permanent dentition [14,15,16,27,28].

The categorical CPI models fitted the data significantly better than the ordinal trend models in both strata. Although the ordinal analyses confirmed a progressive increase in expected DMF-T counts, they assumed an equivalent relative change from CPI 0 to CPI 1 and from CPI 1 to CPI 2. The superior fit of the categorical models indicates that this assumption was not fully supported. CPI should therefore be retained as a categorical screening variable in the principal interpretation rather than represented exclusively by a single linear trend coefficient.

Age was not statistically associated with DMF-T after adjustment within either stratum, and neither a quadratic age term nor CPI-by-continuous-age interaction was significant. This does not imply that dental development or cumulative exposure time is irrelevant. Within the 6–12-year stratum, participants likely differed substantially in the number of erupted permanent teeth contributing to DMF-T and the number of sextants available for CPI assessment. Because these quantities were not retained, chronological age could not fully represent dentition stage, and part of the CPI–DMF-T gradient may reflect unequal measurement opportunities for both exposure and outcome.

The microbiological interpretation also requires caution. Dental caries is associated with ecological changes in the oral biofilm and with alterations in microbial composition and function rather than with the presence of a single microorganism. Systematic reviews have identified differences between the oral microbiota of caries-free and caries-affected individuals, including adolescents, but have also reported substantial methodological and biological heterogeneity [41,42]. Plaque capable of producing bleeding on probing or gingival inflammation is therefore not necessarily equivalent to a cariogenic biofilm. The present association is more plausibly explained by shared behavioral and ecological conditions than by identical pathogenic mechanisms.

The high prevalence of caries experience must be interpreted in relation to recruitment. Participants were consecutive patients attending dental services for examination or treatment rather than members of a probability-based population sample. Care-seeking children and adolescents may have greater symptoms, treatment needs, or previous restorative experience than the general population. Selection bias may therefore have increased both the observed prevalence and the strength of some associations. The results describe relationships within this clinical sample and cannot be generalized to all children and adolescents in Southwest Romania.

From a clinical perspective, unfavorable CPI findings should not be used as a caries prediction tool. In school dental services, however, bleeding or calculus identified during screening can prompt a broader assessment that includes direct caries examination, plaque control, dietary sugar and sweetened-beverage exposure, fluoride use, toothbrushing, and dental-attendance patterns. School protocols could integrate gingival screening with caries assessment, supervised toothbrushing, fluoride-based prevention, dietary counseling, and referral for unmet restorative needs [43,44,45,46,47,48,49,50,51,52,53,54,55,56].

Taken together, higher CPI categories were associated with greater cumulative caries experience in this clinical sample. The attenuation after PI adjustment is consistent with substantial overlap between contemporaneous CPI and plaque measurements. Unfavorable CPI findings may prompt a comprehensive oral-health assessment, but the present study does not establish CPI as an independent causal determinant, a risk marker with validated predictive performance, or a measure of future caries susceptibility.

### Strengths and Limitations

The study included a relatively large clinical sample recruited from four dental care settings in three counties. Examinations followed a standardized protocol and were performed by one trained examiner. The analyses separated prevalence from count outcomes, formally tested age-related effect modification, evaluated PI and GI in separate models, and included generalized Poisson sensitivity analyses and distributional diagnostics.

Several limitations must be considered. First, the cross-sectional design cannot establish the temporal sequence among plaque accumulation, gingival inflammation, CPI findings, and caries development. Second, consecutive non-probability recruitment from dental care settings introduces selection bias and limits generalizability. The very high caries prevalence is compatible with a care-seeking clinical sample and should not be extrapolated to the regional pediatric population.

Recruitment-center identifiers, center-specific screening logs, and individual exclusion reasons were not retained in the analytical database. The models could therefore adjust for county but not distinguish the university clinic from the school dental office in Dolj County, and participant flow could not be reported by center. Differences in referral patterns, reasons for attendance, treatment needs, and previous restorations may have produced residual center-related confounding.

The number of fully erupted permanent teeth and the number of CPI-eligible sextants were not retained as participant-level analytical variables. Participants were therefore not fully comparable in the number of teeth contributing to DMF-T or the number of sites at which a higher CPI category could be detected. This shared measurement-opportunity problem is particularly important in the mixed-dentition stratum and may partly account for the observed gradient. DMF-T was not analytically capped; the observed maximum of 10 arose naturally. Although third molars were excluded from CPI assessment, their contribution to the final participant-level DMF-T variable could not be verified from the analytical database. The D, M, and F components were not retained separately, so the analysis cannot determine whether the association primarily reflected untreated disease, previous treatment, or missing teeth. Radiographs were not used, which may have reduced detection of proximal and early lesions.

Sixth, CPI is a screening index and does not provide a complete periodontal diagnosis or detailed information about the extent and distribution of gingival findings. Studies using CPI in adolescents have shown that bleeding and calculus may be influenced by demographic, behavioral, and contextual factors [37,40]. Seventh, Gingival Index and Plaque Index were calculated as participant-level mean scores, but the underlying tooth-level and surface-level values were not retained as separate analytical variables. This prevented evaluation of their intraoral distribution and limited more detailed assessment of measurement variability.

Index-specific repeat-examination data were not retained, and weighted kappa for CPI and absolute-agreement intraclass correlation coefficients for DMF-T, PI, and GI could not be calculated. The historical pooled kappa is therefore not evidence that each index was reproducible. In addition, the adjusted models did not include sugar intake, sweetened beverages, fluoride exposure, toothbrushing practices, parental education, socioeconomic circumstances, or previous dental attendance. Residual confounding by these common determinants may be substantial, and the association should not be described as independent after comprehensive confounder control.

The Poisson models showed substantial underdispersion. Generalized Poisson sensitivity analyses confirmed the primary CPI associations but also showed that statistical significance in the PI-adjusted adolescent models depended on distributional specification. The contextual findings should therefore be interpreted as model-sensitive, even though the direction and magnitude of the point estimates were broadly similar.

Plaque Index and Gingival Index were highly correlated and were therefore evaluated in separate contextual models. The attenuation of the CPI estimates after Plaque Index adjustment indicates overlapping clinical information but does not establish mediation or causality.

## 5. Conclusions

In this consecutively recruited clinical sample from Southwest Romania, higher CPI screening categories were associated with greater permanent-dentition DMF-T experience. Primary expected-count estimates remained strong in generalized Poisson sensitivity analyses, and there was no statistical evidence that the association differed by age stratum or continuous age.

PI adjustment markedly attenuated CPI estimates, consistent with overlap between contemporaneous plaque and CPI findings. Evidence for an additional CPI association after PI adjustment was sensitive to model specification, whereas GI-adjusted associations were more persistent. These findings do not establish causality, mediation, or predictive validity.

Interpretation is further limited by clinical recruitment, unmeasured behavioral and socioeconomic confounders, unavailable center-level identifiers, lack of index-specific reliability estimates, and absence of participant-level counts of erupted permanent teeth and CPI-eligible sextants. Unequal measurement opportunities may therefore explain part of the observed gradient. Representative longitudinal studies with complete dentition-stage, center, D/M/F, and reliability data are required before CPI can be evaluated as an additional predictor of future caries.

## Figures and Tables

**Table 1:** Demographic and clinical characteristics by age stratum.

Characteristic	Age 6–12 Years (n = 407)	Age 13–19 Years (n = 231)
Age, years	9.64 ± 1.78; median 10 (IQR 3); 6–12	14.64 ± 1.52; median 14 (IQR 3); 13–19
Girls, n (%)	218 (53.6)	119 (51.5)
Boys, n (%)	189 (46.4)	112 (48.5)
Urban residence, n (%)	245 (60.2)	128 (55.4)
Rural residence, n (%)	162 (39.8)	103 (44.6)
Dolj/Gorj/Olt, n	131/146/130	82/67/82
Caries index	DMF-T 4.71 ± 2.55; median 5 (IQR 4); 0–10	DMF-T 4.93 ± 2.58; median 5 (IQR 4); 0–10
PI	1.78 ± 0.79; median 1.75 (IQR 1.49); 0.20–3.15	1.84 ± 0.85; median 1.90 (IQR 1.60); 0.25–3.15
GI	1.35 ± 0.72; median 1.39 (IQR 1.27); 0.10–2.80	1.38 ± 0.73; median 1.39 (IQR 1.37); 0.10–2.74
CPI 0/1/2, n (%)	127 (31.2)/156 (38.3)/124 (30.5)	76 (32.9)/72 (31.2)/83 (35.9)
Caries experience present, n (%)	384 (94.3)	223 (96.5)

Note. Values are presented as mean ± standard deviation, median (IQR), range, or n (%), as appropriate. DMF-T, Decayed, Missing, and Filled Teeth index; CPI, Community Periodontal Index; PI, Plaque Index; GI, Gingival Index.

**Table 2:** Prevalence of caries experience and DMF-T burden across CPI categories by age stratum.

Age	CPI	n	Caries Experience Present, n (%)	95% Wilson CI, %	Mean ± SD	Median (IQR)	Range
6–12	0	127	105 (82.7)	75.2–88.3	2.20 ± 1.71	2 (2)	0–7
6–12	1	156	155 (99.4)	96.5–99.9	4.99 ± 1.93	5 (2)	0–10
6–12	2	124	124 (100.0)	97.0–100.0	6.93 ± 1.48	7 (2)	4–10
13–19	0	76	68 (89.5)	80.6–94.6	2.32 ± 1.57	2 (2)	0–7
13–19	1	72	72 (100.0)	94.9–100.0	5.33 ± 1.99	5 (3)	1–10
13–19	2	83	83 (100.0)	95.6–100.0	6.98 ± 1.51	7 (2)	3–10

**Table 3:** Correlation and Kruskal–Wallis results by age stratum.

Age	Caries–CPI rho (95% CI; *p*)	Caries–PI rho (95% CI; *p*)	Caries–GI rho (95% CI; *p*)	Caries–Age rho (95% CI; *p*)	H(2)	Rank Epsilon-Squared (95% CI)
6–12	0.743 (0.693–0.787; <0.001)	0.880 (0.856–0.898; <0.001)	0.847 (0.820–0.869; <0.001)	0.003 (−0.093–0.098; 0.945)	225.8	0.556 (0.509–0.625)
13–19	0.749 (0.686–0.801; <0.001)	0.876 (0.843–0.902; <0.001)	0.837 (0.794–0.866; <0.001)	−0.088 (−0.211–0.036; 0.181)	132.1	0.574 (0.499–0.652)

**Table 4:** Primary categorical Poisson models for age-stratified DMF-T burden.

Age Stratum	Predictor	Expected Count Ratio (95% CI)	*p*
6–12	CPI 1 vs. 0	2.27 (1.98–2.61)	<0.001
6–12	CPI 2 vs. 0	3.16 (2.76–3.62)	<0.001
6–12	Age, per year	0.99 (0.97–1.02)	0.484
6–12	Male vs. female	1.05 (0.96–1.15)	0.287
6–12	Urban vs. rural	1.03 (0.94–1.13)	0.497
6–12	Gorj vs. Dolj	1.06 (0.95–1.18)	0.303
6–12	Olt vs. Dolj	1.00 (0.89–1.12)	0.951
13–19	CPI 1 vs. 0	2.28 (1.91–2.73)	<0.001
13–19	CPI 2 vs. 0	2.99 (2.53–3.56)	<0.001
13–19	Age, per year	1.00 (0.96–1.04)	0.978
13–19	Male vs. female	0.98 (0.87–1.10)	0.707
13–19	Urban vs. rural	1.07 (0.95–1.20)	0.290
13–19	Gorj vs. Dolj	1.01 (0.87–1.17)	0.898
13–19	Olt vs. Dolj	0.95 (0.83–1.10)	0.505

CI, confidence interval. Exponentiated coefficients are interpreted as ratios of expected DMF-T counts. Models were adjusted for age, sex, area of residence, and county.

**Table 5:** Contextual Poisson models including Plaque Index or Gingival Index.

Age	Model	Term	Expected Count Ratio (95% CI)	*p*	AIC
6–12	PI model	PI, per 0.5 unit	1.35 (1.28–1.42)	<0.001	1522
6–12	PI model	CPI 1 vs. 0	1.31 (1.11–1.55)	0.002	
6–12	PI model	CPI 2 vs. 0	1.17 (0.94–1.45)	0.169	
6–12	GI model	GI, per 0.5 unit	1.30 (1.24–1.38)	<0.001	1555
6–12	GI model	CPI 1 vs. 0	1.45 (1.23–1.71)	<0.001	
6–12	GI model	CPI 2 vs. 0	1.43 (1.16–1.77)	<0.001	
13–19	PI model	PI, per 0.5 unit	1.29 (1.21–1.38)	<0.001	874.6
13–19	PI model	CPI 1 vs. 0	1.26 (0.99–1.60)	0.058	
13–19	PI model	CPI 2 vs. 0	1.26 (0.96–1.66)	0.101	
13–19	GI model	GI, per 0.5 unit	1.27 (1.18–1.36)	<0.001	892.2
13–19	GI model	CPI 1 vs. 0	1.50 (1.21–1.87)	<0.001	
13–19	GI model	CPI 2 vs. 0	1.54 (1.19–1.99)	0.001	

All contextual models were additionally adjusted for age, sex, area of residence, and county. Pearson chi-square/df ranged from 0.42 to 0.53, indicating underdispersion. PI and GI were modeled separately because of their high correlation. AIC is reported once per model.

## Data Availability

The data supporting the findings of this study are available from the corresponding authors upon reasonable request.

## Supplementary Materials

The following supporting information can be downloaded at: https://www.mdpi.com/article/10.3390/children13081060/s1, Table S1: Complete contextual Poisson models including all adjusted covariates; Table S2: Generalized Poisson sensitivity analyses permitting underdispersion; Figure S1: Distribution of permanent-dentition DMF-T scores by age stratum; Figure S2: Jittered scatterplots of DMF-T and Plaque Index by age stratum. Lines are descriptive linear trends; Figure S3: Jittered scatterplots of DMF-T and Gingival Index by age stratum. Lines are descriptive linear trends; Figure S4: Overall participant flow. Center-specific counts and individual exclusion reasons were not retained in the analytical database.

## References

[1] World Health Organization (2022). Global Oral Health Status Report: Towards Universal Health Coverage for Oral Health by 2030.

[2] Peres M.A., Macpherson L.M.D., Weyant R.J., Daly B., Venturelli R., Mathur M.R., Listl S., Celeste R.K., Guarnizo-Herreño C.C., Kearns C. (2019). Oral diseases: A global public health challenge. Lancet.

[3] Wen P.Y.F., Chen M.X., Zhong Y.J., Dong Q.Q., Wong H.M. (2022). Global burden and inequality of dental caries, 1990 to 2019. J. Dent. Res..

[4] Kassebaum N.J., Bernabe E., Dahiya M., Bhandari B., Murray C.J.L., Marcenes W. (2015). Global burden of untreated caries: A systematic review and metaregression. J. Dent. Res..

[5] Uribe S.E., Innes N., Maldupa I. (2021). The global prevalence of early childhood caries: A systematic review with meta-analysis using the WHO diagnostic criteria. Int. J. Paediatr. Dent..

[6] Grieshaber A., Waltimo T., Haschemi A.A., Bornstein M.M., Kulik E.M. (2024). Dental caries and associated factors in 7-, 12- and 15-year-old schoolchildren in Basel-Landschaft, Switzerland. Int. J. Paediatr. Dent..

[7] Sabbagh S., Mohammadi-Nasrabadi F., Ravaghi V., Azadi Mood K., Sarraf Shirazi A., Abedi A.S., Noorollahian H. (2023). Food insecurity and dental caries prevalence in children and adolescents: A systematic review and meta-analysis. Int. J. Paediatr. Dent..

[8] Moynihan P.J., Kelly S.A.M. (2014). Effect on caries of restricting sugars intake: Systematic review to inform WHO guidelines. J. Dent. Res..

[9] Moynihan P. (2016). Sugars and dental caries: Evidence for setting a recommended threshold for intake. Adv. Nutr..

[10] Walsh T., Worthington H.V., Glenny A.M., Marinho V.C.C., Jeroncic A. (2019). Fluoride toothpastes of different concentrations for preventing dental caries. Cochrane Database Syst. Rev..

[11] Khalid G., Metzner F., Pawils S. (2022). Prevalence of dental neglect and associated risk factors in children and adolescents: A systematic review. Int. J. Paediatr. Dent..

[12] Knorst J.K., Tomazoni F., Sfreddo C.S., Vettore M.V., Hesse D., Ardenghi T.M. (2022). Social capital and oral health in children and adolescents: A systematic review and meta-analysis. Community Dent. Oral Epidemiol..

[13] Chapple I.L.C., Bouchard P., Cagetti M.G., Campus G., Carra M.C., Cocco F., Nibali L., Hujoel P., Laine M.L., Lingström P. (2017). Interaction of lifestyle, behaviour or systemic diseases with dental caries and periodontal diseases: Consensus report of group 2 of the joint EFP/ORCA workshop on the boundaries between caries and periodontal diseases. J. Clin. Periodontol..

[14] Trombelli L., Farina R., Silva C.O., Tatakis D.N. (2018). Plaque-induced gingivitis: Case definition and diagnostic considerations. J. Clin. Periodontol..

[15] Murakami S., Mealey B.L., Mariotti A., Chapple I.L.C. (2018). Dental plaque-induced gingival conditions. J. Clin. Periodontol..

[16] World Health Organization (2013). Oral Health Surveys: Basic Methods.

[17] Baciu D., Danila I., Balcos C., Gallagher J.E., Bernabé E. (2015). Caries experience among Romanian schoolchildren: Prevalence and trends 1992–2011. Community Dent. Health.

[18] Sfeatcu R., Cărămidă M., Sava-Rosianu R., Matichescu M.L., Galuscan A., Dumitrache M.A. (2023). Carious status and socio-behavioral risk factors among 12-year-old children in the South-Central region of Romania. BMC Oral Health.

[19] Dumitrescu R., Sava-Rosianu R., Jumanca D., Balean O., Damian L.R., Campus G.G., Maricutoiu L., Alexa V.T., Sfeatcu R., Daguci C. (2022). Dental caries, oral health behavior, and living conditions in 6-8-year-old Romanian school children. Children.

[20] Tudoroniu C., Popa M., Iacob S.M., Pop A.L., Năsui B.A. (2020). Correlation of caries prevalence, oral health behavior and sweets nutritional habits among 10- to 19-year-old Cluj-Napoca Romanian adolescents. Int. J. Environ. Res. Public Health.

[21] Sava-Rosianu R., Campus G., Matichescu A., Balean O., Dumitrache M.A., Lucaciu P.O., Daguci L., Barlean M.C., Maricutoiu L., Postolache M. (2021). Caries prevalence associated with oral health-related behaviors among Romanian schoolchildren. Int. J. Environ. Res. Public Health.

[22] Schulze Z.J., Schubert F., Gernhardt C.R., Krayl N., Peters A., Unverzagt S., Wagner K., Wienke A., Führer A. (2025). Area-level factors of dental caries in children and adolescents in European neighborhoods: A systematic review. J. Urban Health.

[23] Lucaciu P.O., Mester A., Constantin I., Orban N., Cosma L., Candrea S., Sava-Rosianu R., Mesaros A.S. (2020). A WHO Pathfinder survey of dental caries in 6- and 12-year-old Transylvanian children and the possible correlation with their family background, oral-health behavior, and the intake of sweets. Int. J. Environ. Res. Public Health.

[24] Funieru C., Klinger A., Băicuș C., Funieru E., Dumitriu H.T., Dumitriu A. (2017). Epidemiology of gingivitis in schoolchildren in Bucharest, Romania: A cross-sectional study. J. Periodontal Res..

[25] Lile I.E., Cojocariu C., Marian D., Hosszu T., Stana A.H., Stana O. (2025). Oral hygiene and dietary behaviors among Romanian schoolchildren: A cross-sectional study. Children.

[26] von Elm E., Altman D.G., Egger M., Pocock S.J., Gotzsche P.C., Vandenbroucke J.P., STROBE Initiative (2007). The Strengthening the Reporting of Observational Studies in Epidemiology (STROBE) statement: Guidelines for reporting observational studies. Lancet.

[27] Loe H., Silness J. (1963). Periodontal disease in pregnancy. I. Prevalence and severity. Acta Odontol. Scand..

[28] Silness J., Loe H. (1964). Periodontal disease in pregnancy. II. Correlation between oral hygiene and periodontal condition. Acta Odontol. Scand..

[29] El Tantawi M., Folayan M.O., Oginni O., Adeniyi A.A., Mapayi B., Yassin R., Chukwumah N.M., Sam-Agudu N.A. (2021). Association between mental health, caries experience and gingival health of adolescents in sub-urban Nigeria. BMC Oral Health.

[30] Aly N.M., Mohamed A.A., Abdelaziz W.E. (2020). Parenting practices and oral health status in rural areas in Egypt: A household survey. BMC Oral Health.

[31] Kaczmarek U., Wrzyszcz-Kowalczyk A., Jankowska K., Prosciak K., Mysiak-Debska M., Przywitowska I., Makulska I. (2020). Oral health conditions in children with idiopathic nephrotic syndrome: A cross-sectional study. BMC Oral Health.

[32] Davidovich E., Grender J., Zini A. (2020). Factors associated with dental plaque, gingivitis, and caries in a pediatric population: A records-based cross-sectional study. Int. J. Environ. Res. Public Health.

[33] Zhang M., Lan J., Zhang T., Sun W., Liu P., Wang Z. (2021). Oral health and caries/gingivitis-associated factors of adolescents aged 12-15 in Shandong province, China: A cross-sectional Oral Health Survey. BMC Oral Health.

[34] Pan Y., Ma Y., Gui Z., Jin Y., Pan J., Yang C., Huang J. (2025). Dental caries, gingivitis and oral health-related quality of life in 12-year-old children. BMC Oral Health.

[35] Kukletova M., Izakovicova Holla L., Musilova K., Broukal Z., Kukla L. (2012). Relationship between gingivitis severity, caries experience and orthodontic anomalies in 13–15-year-old adolescents in Brno, Czech Republic. Community Dent. Health.

[36] Gopinath V.K., Rahman B., Awad M.A. (2015). Assessment of gingival health among school children in Sharjah, United Arab Emirates. Eur. J. Dent..

[37] Chang P.S., Huang C.J., Hsiang C.L., Lai H., Tsai A.I. (2021). Prevalence of dental caries and periodontal disease of high school students aged 15 to 18 years in Taiwan. Int. J. Environ. Res. Public Health.

[38] Bashirian S., Shirahmadi S., Seyedzadeh-Sabounchi S., Soltanian A.R., Karimi-Shahanjarini A., Vahdatinia F. (2018). Association of caries experience and dental plaque with sociodemographic characteristics in elementary school-aged children: A cross-sectional study. BMC Oral Health.

[39] Milona M., Janiszewska-Olszowska J., Szmidt M., Kłoda K., Olszowski T. (2021). Oral health related behaviors in relation to DMFT indexes of teenagers in an urban area of North-West Poland: Dental caries is still a common problem. Int. J. Environ. Res. Public Health.

[40] Zhan J., Zhang Y., Wang X., Tai B., Hu D., Lin H., Wang B., Si Y., Wang C., Zheng S. (2021). Related factors of periodontal health among Chinese middle school students: Findings from a national cross-sectional survey. BMC Oral Health.

[41] Veenman F., van Dijk A., Arredondo A., Medina-Gomez C., Wolvius E., Rivadeneira F., Àlvarez G., Blanc V., Kragt L. (2024). Oral microbiota of adolescents with dental caries: A systematic review. Arch. Oral Biol..

[42] Pagnussatti M.E.L., de Barros Santos H.S., Parolo C.C.F., Hilgert J.B., Arthur R.A. (2024). Oral microbiota: Taxonomic composition and functional profile in caries-free and in caries-affected individuals—A systematic review. Arch. Oral Biol..

[43] American Academy of Pediatric Dentistry (2025). Caries-risk assessment and management for infants, children, and adolescents. The Reference Manual of Pediatric Dentistry.

[44] World Health Organization (2020). Ending Childhood Dental Caries: WHO Implementation Manual.

[45] Rojas-Briceno N.B., Oc Carrasco O.J., Silva Diaz Y.A., Ramírez C.M.O., Salazar O.P., Tuesta-Mendoza S.J., Silva-López J.O. (2024). Knowledge and attitudes of parents about oral health in the primary dentition stage in a Peruvian High Andean City. Int. J. Environ. Res. Public Health.

[46] Park Y.H., Choi Y.Y. (2022). Feeding practices and early childhood caries in Korean preschool children. Int. Dent. J..

[47] Clark M.B., Braun P.A. (2021). Promotion of oral health and prevention of dental caries among children in primary care. JAMA.

[48] Weyant R.J., Tracy S.L., Anselmo T.T., Beltrán-Aguilar E.D., Donly K.J., Frese W.A., Hujoel P.P., Iafolla T., Kohn W., Kumar J. (2013). Topical fluoride for caries prevention: Executive summary of updated clinical recommendations and supporting systematic review. J. Am. Dent. Assoc..

[49] Iheozor-Ejiofor Z., Walsh T., Lewis S.R., Riley P., Boyers D., Clarkson J.E., Worthington H.V., Glenny A.M., O’Malley L. (2024). Water fluoridation for the prevention of dental caries. Cochrane Database Syst. Rev..

[50] Marinho V.C.C., Worthington H.V., Walsh T., Clarkson J.E. (2013). Fluoride varnishes for preventing dental caries in children and adolescents. Cochrane Database Syst. Rev..

[51] Akera P., Kennedy S.E., Lingam R., Obwolo M.J., Schutte A.E., Richmond R. (2022). Effectiveness of primary school-based interventions in improving oral health of children in low- and middle-income countries: A systematic review and meta-analysis. BMC Oral Health.

[52] Das H., Janakiram C., S V.K., Karuveettil V. (2025). Effectiveness of school-based oral health education interventions on oral health status and oral hygiene behaviors among schoolchildren: An umbrella review. Evid. Based Dent..

[53] Aliakbari E., Gray-Burrows K.A., Vinall-Collier K.A., Edwebi S., Marshman Z., McEachan R.R.C., Day P.F. (2021). Home-based toothbrushing interventions for parents of young children to reduce dental caries: A systematic review. Int. J. Paediatr. Dent..

[54] Stein C., Santos N.M.L., Hilgert J.B., Hugo F.N. (2018). Effectiveness of oral health education on oral hygiene and dental caries in schoolchildren: Systematic review and meta-analysis. Community Dent. Oral Epidemiol..

[55] Dos Santos A.P.P., de Oliveira B.H., Nadanovsky P. (2018). A systematic review of the effects of supervised toothbrushing on caries incidence in children and adolescents. Int. J. Paediatr. Dent..

[56] Kumar S., Tadakamadla J., Johnson N.W. (2016). Effect of toothbrushing frequency on incidence and increment of dental caries: A systematic review and meta-analysis. J. Dent. Res..

