# Supplementary material for: Age-Stratified Association Between CPI Screening Categories and Caries Experience in the Permanent Dentition of Children and Adolescents from Southwest Romania: A Multicenter Cross-Sectional Clinical Study
Source: Children (Basel). 2026 Aug 10;13(8):1060. doi: 10.3390/children13081060 (PMC13510733; doi:10.3390/children13081060)
Supplement: Supplementary file 1 [file children-13-01060-s001.zip › children-4464446-supplementary.pdf]

children

Supplementary Material

Age-Stratified Association Between CPI Screening Categories and Caries Experience in the Permanent Dentition of Children and Adolescents from Southwest Romania

Table S1. Complete contextual Poisson models including all adjusted covariates

Age	Model	Term	Expected count ratio	95% CI	p	AIC
6–12	PI model	CPI 1 vs 0	1.31	1.11–1.55	0.002	1521.8
6–12	PI model	CPI 2 vs 0	1.17	0.94–1.45	0.169	1521.8
6–12	PI model	Male vs female	1.04	0.95–1.14	0.361	1521.8
6–12	PI model	Urban vs rural	0.99	0.90–1.09	0.838	1521.8
6–12	PI model	Gorj vs Dolj	1.04	0.93–1.15	0.531	1521.8
6–12	PI model	Olt vs Dolj	0.97	0.87–1.09	0.611	1521.8
6–12	PI model	Age, per year	1.00	0.97–1.02	0.867	1521.8
6–12	PI model	PI, per 0.5 unit	1.35	1.28–1.42	<0.001	1521.8
6–12	GI model	CPI 1 vs 0	1.45	1.23–1.71	<0.001	1554.5
6–12	GI model	CPI 2 vs 0	1.43	1.16–1.76	<0.001	1554.5
6–12	GI model	Male vs female	1.05	0.96–1.15	0.326	1554.5
6–12	GI model	Urban vs rural	1.00	0.92–1.10	0.925	1554.5
6–12	GI model	Gorj vs Dolj	1.03	0.93–1.15	0.567	1554.5
6–12	GI model	Olt vs Dolj	0.94	0.84–1.06	0.326	1554.5
6–12	GI model	Age, per year	0.99	0.97–1.02	0.609	1554.5
6–12	GI model	GI, per 0.5 unit	1.30	1.24–1.38	<0.001	1554.5
13–19	PI model	CPI 1 vs 0	1.26	0.99–1.59	0.058	874.6
13–19	PI model	CPI 2 vs 0	1.26	0.96–1.66	0.101	874.6
13–19	PI model	Male vs female	0.93	0.83–1.05	0.252	874.6
13–19	PI model	Urban vs rural	1.03	0.91–1.16	0.651	874.6
13–19	PI model	Gorj vs Dolj	1.01	0.88–1.17	0.879	874.6
13–19	PI model	Olt vs Dolj	1.01	0.88–1.16	0.906	874.6
13–19	PI model	Age, per year	1.01	0.97–1.05	0.604	874.6
13–19	PI model	PI, per 0.5 unit	1.29	1.21–1.38	<0.001	874.6
13–19	GI model	CPI 1 vs 0	1.50	1.21–1.87	<0.001	892.2
13–19	GI model	CPI 2 vs 0	1.54	1.19–1.99	0.001	892.2
13–19	GI model	Male vs female	0.96	0.86–1.08	0.536	892.2
13–19	GI model	Urban vs rural	1.02	0.91–1.15	0.722	892.2
13–19	GI model	Gorj vs Dolj	1.02	0.88–1.17	0.836	892.2
13–19	GI model	Olt vs Dolj	1.02	0.88–1.17	0.802	892.2
13–19	GI model	Age, per year	1.02	0.98–1.06	0.415	892.2
13–19	GI model	GI, per 0.5 unit	1.27	1.18–1.36	<0.001	892.2

Note. AIC is repeated here to facilitate model identification. PI and GI effects are expressed per 0.5-unit increase. All models include age, sex, residence, and county.

Table S2. Generalized Poisson sensitivity analyses permitting underdispersion

Age	Model	Term	Expected count ratio	95% CI	p	α (95% CI)
6–12	Primary	CPI 1 vs 0	2.20	1.95–2.48	<0.001	−0.107 (−0.164 to −0.049)
6–12	Primary	CPI 2 vs 0	3.04	2.69–3.43	<0.001	−0.107 (−0.164 to −0.049)
6–12	PI-adjusted	CPI 1 vs 0	1.23	1.12–1.35	<0.001	−0.357 (−0.401 to −0.313)
6–12	PI-adjusted	CPI 2 vs 0	1.09	0.97–1.24	0.145	−0.357 (−0.401 to −0.313)
6–12	PI-adjusted	PI per 0.5 unit	1.34	1.30–1.39	<0.001	−0.357 (−0.401 to −0.313)
6–12	GI-adjusted	CPI 1 vs 0	1.35	1.22–1.51	<0.001	−0.285 (−0.332 to −0.237)
6–12	GI-adjusted	CPI 2 vs 0	1.32	1.16–1.52	<0.001	−0.285 (−0.332 to −0.237)
6–12	GI-adjusted	GI per 0.5 unit	1.31	1.26–1.36	<0.001	−0.285 (−0.332 to −0.237)
13–19	Primary	CPI 1 vs 0	2.19	1.90–2.54	<0.001	−0.170 (−0.241 to −0.098)
13–19	Primary	CPI 2 vs 0	2.85	2.48–3.28	<0.001	−0.170 (−0.241 to −0.098)
13–19	PI-adjusted	CPI 1 vs 0	1.21	1.06–1.38	0.005	−0.365 (−0.421 to −0.309)
13–19	PI-adjusted	CPI 2 vs 0	1.22	1.04–1.42	0.013	−0.365 (−0.421 to −0.309)
13–19	PI-adjusted	PI per 0.5 unit	1.28	1.24–1.34	<0.001	−0.365 (−0.421 to −0.309)
13–19	GI-adjusted	CPI 1 vs 0	1.43	1.24–1.64	<0.001	−0.311 (−0.373 to −0.250)
13–19	GI-adjusted	CPI 2 vs 0	1.45	1.24–1.71	<0.001	−0.311 (−0.373 to −0.250)
13–19	GI-adjusted	GI per 0.5 unit	1.27	1.21–1.33	<0.001	−0.311 (−0.373 to −0.250)

Note. Negative alpha values indicate underdispersion. Models were fitted using Python 3.13.5 and statsmodels 0.14.6.

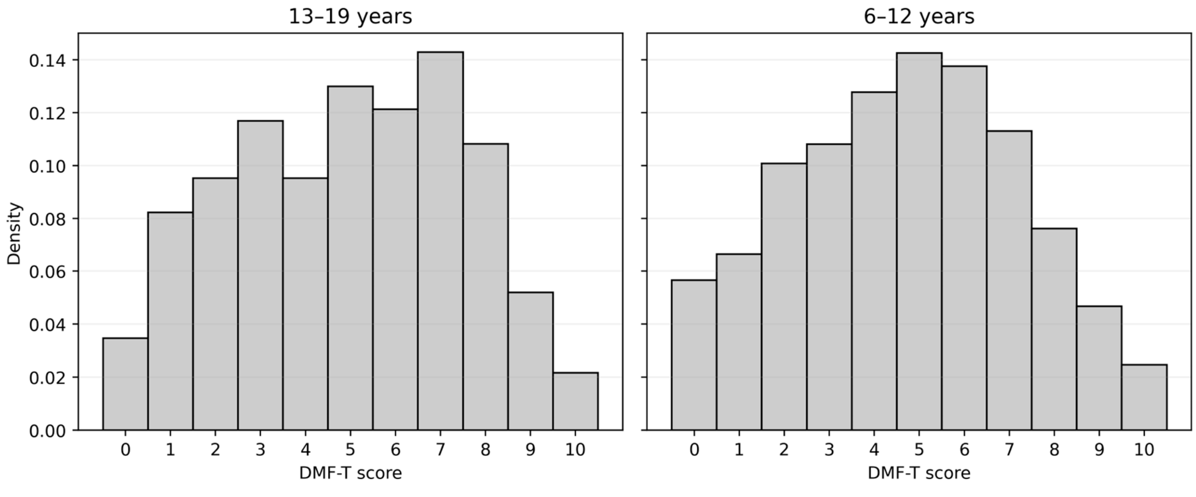

Figure S1. Distribution of permanent-dentition DMF-T scores by age stratum.

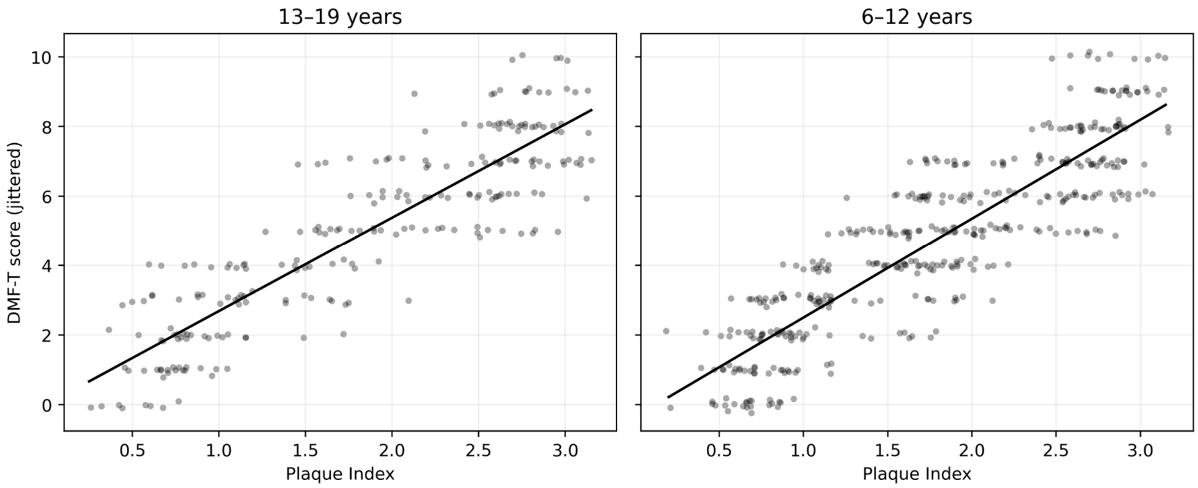

Figure S2. Jittered scatterplots of DMF-T and Plaque Index by age stratum. Lines are descriptive linear trends.

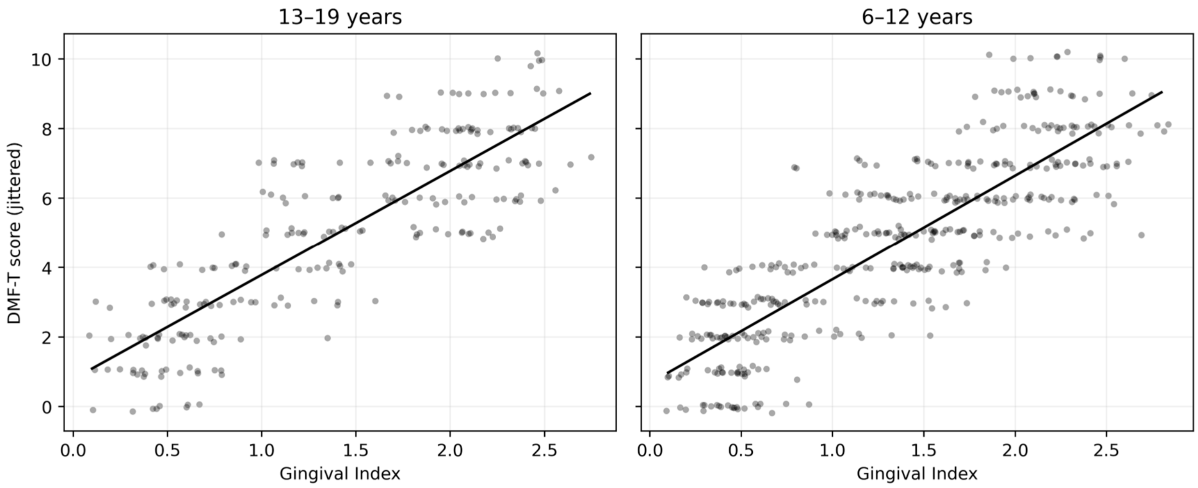

Figure S3. Jittered scatterplots of DMF-T and Gingival Index by age stratum. Lines are descriptive linear trends.

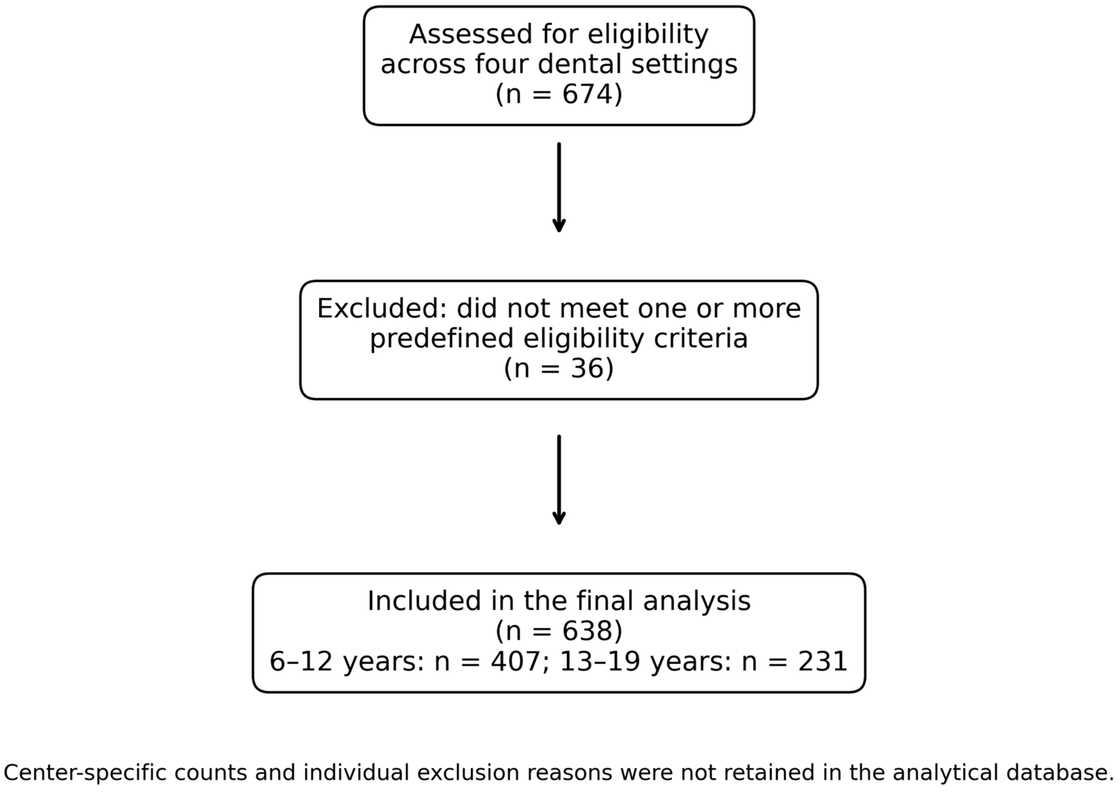

Figure S4. Overall participant flow. Center-specific counts and individual exclusion reasons were not retained in the analytical database.
